# Penetrating thoracic stab wounds and the cardiac box: a single-center experience of in-hospital treatment and outcome in Germany

**DOI:** 10.1186/s13049-026-01555-y

**Published:** 2026-01-17

**Authors:** Sebastian M. Rabe, Uwe Scheuermann, Suzanne Zeidler, Christian Kleber, Matthias Steinert, Sebastian Krämer

**Affiliations:** 1 Department of Visceral, Transplant, Thoracic and Vascular Surgery, University Hospital of Leipzig, Liebigstr. 20, Leipzig, D-04103 Germany; 2 Department of Orthopaedic, Trauma and Plastic Surgery, University Hospital of Leipzig, Liebigstr. 20, Leipzig, D-04103 Germany

**Keywords:** Thoracic stab injury, Cardiac box, Penetrating cardiac injury

## Abstract

**Background:**

Thoracic stab injuries (TSI) are rare but potentially life-threatening emergencies. In Germany, their incidence in emergency departments remains low. The *cardiac box* (CB) concept has been proposed to identify cardiac involvement in penetrating thoracic trauma, although its clinical relevance remains uncertain. This study aimed to evaluate the in-hospital management of TSI and to assess the predictive value of the cardiac box for major intrathoracic injuries.

**Methods:**

A retrospective and exploratory analysis was conducted of all patients with TSI resulting from assault or self-harm who were admitted to a certified Level 1 trauma centre between January 2020 and June 2024. Prehospital and in-hospital variables were descriptively analysed.

**Results:**

Fifty-six male patients were included (median age 28.5 years). Sixteen patients sustained injuries within the cardiac box (CB), and thirty-nine outside this area (NCB). All CB patients (100%) and 95% of NCB patients were admitted via the emergency department. The annual proportion of thoracic stab injuries among all emergency presentations ranged from 1.4% to 2.4%.

The median Injury Severity Score (ISS) was significantly higher in the CB group (9.5 vs 3; *p* = 0.045), whereas the distribution of intrathoracic injury types and initial haemodynamic parameters (MAP CB: 93 mmHg vs NCB: 97 mmHg; *p* = 0.925) did not differ significantly. Two patients in the CB group had a cardiac and/or great vessel injury. Two NCB patients received prehospital chest tubes. In the emergency department, chest tubes were placed in 23.2% of patients, with no significant group difference. Median intrahospital transfer time to the target department was shorter in CB patients (38 vs 67 min).

Video-assisted thoracic surgery (VATS) was performed in eight patients (CB: 25%; NCB: 10.3%; *p* = 0.241), and one open procedure was undertaken in each group. Major complications (Clavien–Dindo ≥ II) occurred more frequently among CB patients (50.1% vs 25.7%; *p* = 0.018). The overall mortality rate was 3.6% (two CB patients).

**Conclusions:**

TSI are rare but serious injuries requiring structured, multidisciplinary in-hospital management. Minimally invasive approaches are feasible in haemodynamically stable patients. The low rate of prehospital chest tube placement warrants further evaluation. The cardiac box concept appears overly simplistic, as clinically significant injuries may also occur outside this anatomical region.

**Supplementary Information:**

The online version contains supplementary material available at 10.1186/s13049-026-01555-y.

## Background

Thoracic stab injuries (TSI) frequently present as life-threatening emergencies, underscoring the imperative for comprehensive and multidisciplinary trauma rooms [1]. In recent years, media coverage in Germany has increasingly highlighted incidents of knife-related violence, prompting political debate and, ultimately, the expansion of knife-prohibition zones in public spaces in 2024 [2]. In Saxony alone, a total of 1,373 knife crimes were recorded in 2023, corresponding to an incidence rate of 33.6 per 100,000 inhabitants [3]. This figure represents a 16.2% increase in violent offences in 2023, accompanied by a nationwide rise in knife-related crime. Notably, however, there remains a lack of uniformity in data collection across the criminal investigation offices of the individual federal states.

Despite the reported rise in knife-related assaults, such injuries remain comparatively uncommon in emergency care settings across German hospitals. Data on the in-hospital management of TSI,^1^ in particular, are scarce. A recent study from Schleswig–Holstein demonstrated that, between 2020 and 2024, the overall incidence of stab wounds presenting to emergency departments remained consistently low, with approximately 30% involving thoracic injuries [4]. In contrast, the incidence of stab wounds at the university hospital in Berlin significantly increased in 2024, although this was not adjusted for concurrent population growth [5]. The 2023 annual report of the TraumaRegister DGU® of the German Society for Trauma Surgery documented a stable 4% proportion of penetrating trauma over the past decade [6].

In the prehospital setting, the “cardiac box” concept has been proposed as a means of predicting the likelihood of cardiac injury resulting from penetrating thoracic trauma such as stab or gunshot wounds [7]. The cardiac box is anatomically defined as the area bordered laterally by the two midclavicular or nipple lines, cranially by the clavicular line, and caudally by the costal margins. Wounds occurring within this area are considered to carry a high risk of concomitant cardiac or great vessel injury. In addition to extended focused assessment with sonography for trauma (eFAST), the cardiac box concept is regarded as one of the few prehospital tools available to predict such injuries, thereby influencing treatment strategies such as the “treat and run” approach and the selection of a receiving hospital with cardiothoracic surgical capability [8, 9]. Nevertheless, the clinical utility of the cardiac box remains uncertain. An injury located outside this anatomical region does not exclude a concomitant cardiac lesion, which, depending on the trauma mechanism, may carry an even higher mortality rate [10].

Accordingly, the present study was undertaken to determine the incidence and in-hospital management of TSI treated at a single level 1 trauma centre, and to evaluate the association between injury localisation, treatment modality, and outcome over a five-year period (2020–2024). A further objective was to assess the validity of the cardiac box concept in predicting cardiac and/or great vessel injury. We hypothesised that the cardiac box, as currently defined, does not represent a sufficient predictor of cardiac or major vascular involvement in patients with thoracic stab wounds.

## Methods

This retrospective and exploratory study included all patients with TSI resulting from assault or self-harm who were primarily admitted to, or transferred for initial management at the University Hospital of Leipzig, a supra-regional (Level 1) certified trauma centre, between January 2020 and June 2024. Patients who sustained penetrating thoracic injuries in the context of occupational or traffic accidents were excluded from analysis, as these three cases were predominantly associated with severe blunt thoracic trauma.

Ethical approval for this study was granted by the Ethics Committee of the Medical Faculty of the University of Leipzig (069/25-ek). Prehospital variables —including operational time, prehospital intubation, and the type and length of weapon used—were extracted from emergency service reports and entered into a dedicated database (Microsoft® Excel® for Microsoft 365 MSO, Version 2410 Build 16.0.18129.20100). In-hospital parameters (e.g. age, sex, injury location, associated injuries, laboratory findings, and timing of surgical intervention) were obtained from institutional patient data management systems (COPRA v2.87, COPRA System GmbH, Berlin, Germany; and SAP Deutschland SE & Co.). All data were subsequently integrated into a pseudonymised database, ensuring that no conclusions could be drawn regarding individual cases.

To determine wound localisation, forensic photo documentation was transferred onto a three-dimensional anatomical model. Where such documentation was unavailable, a 3D reconstruction of the chest wall was generated from the patient’s computed tomography (CT) scan (Uniview Sectra, Version 25.1.6.4226, Sectra AB, Linköping, Sweden).

**Fig. 1:**
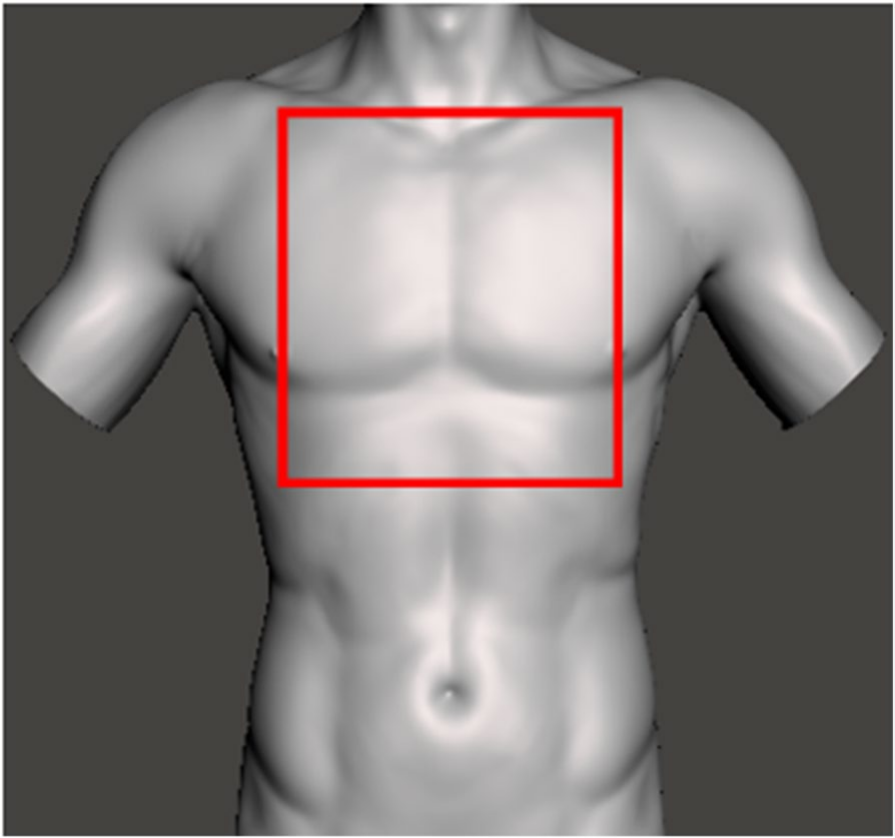
The concept of the cardiac box – an imagined anatomical square between the two midclavicular lines cranially, midclavicular lines laterally, and caudally the ribcage edges. Based on the publication of Siemens et al. [11] and Evans et al. [12]

Patients were categorised according to the anatomical position of the stab wound as either inside or outside the cardiac box (CB or NCB). Since its first introduction in 1967, the borders of the cardiac box vary widely among international studies. We therefore decided to define the borders of the cardiac box according to the publications by Siemens et al. [11] and Evans et al. [12] (Fig. 1):

- Cranial: the line between the midclavicular points of the left and right midclavicular lines
- Lateral: the midclavicular lines
- Caudal: the line between the ribcage point of the midclavicular lines

As no individual presented with injuries involving both regions, each patient could be clearly assigned to one category. Thoracic injuries were identified on the basis of intra-operative findings, radiological imaging, or clinical examination. Cardiac and great vessel injuries were defined as any confirmed or suspected lesion involving the ventricles, atria, aorta, pulmonary artery, or pulmonary veins.

Statistical analyses were conducted using SPSS version 29 (IBM SPSS Statistics, Chicago, IL, USA). Depending on the distribution of variables, data are presented as medians with interquartile ranges (IQR) or as means with standard deviations (SD). Normality was assessed using the Kolmogorov–Smirnov test, and equality of variances with the F-test. For comparisons between groups, Student’s t-test or the Mann–Whitney U-test was applied as appropriate. A p-value < 0.05 was considered statistically significant. Graphical representations were produced using GraphPad Prism (version 10, Boston, MA, USA).

Given the small sample size and multiple subgroup analyses, our findings should be interpreted as exploratory, and the potential risk of type I error cannot be excluded.

## Results

**Fig. 2:**
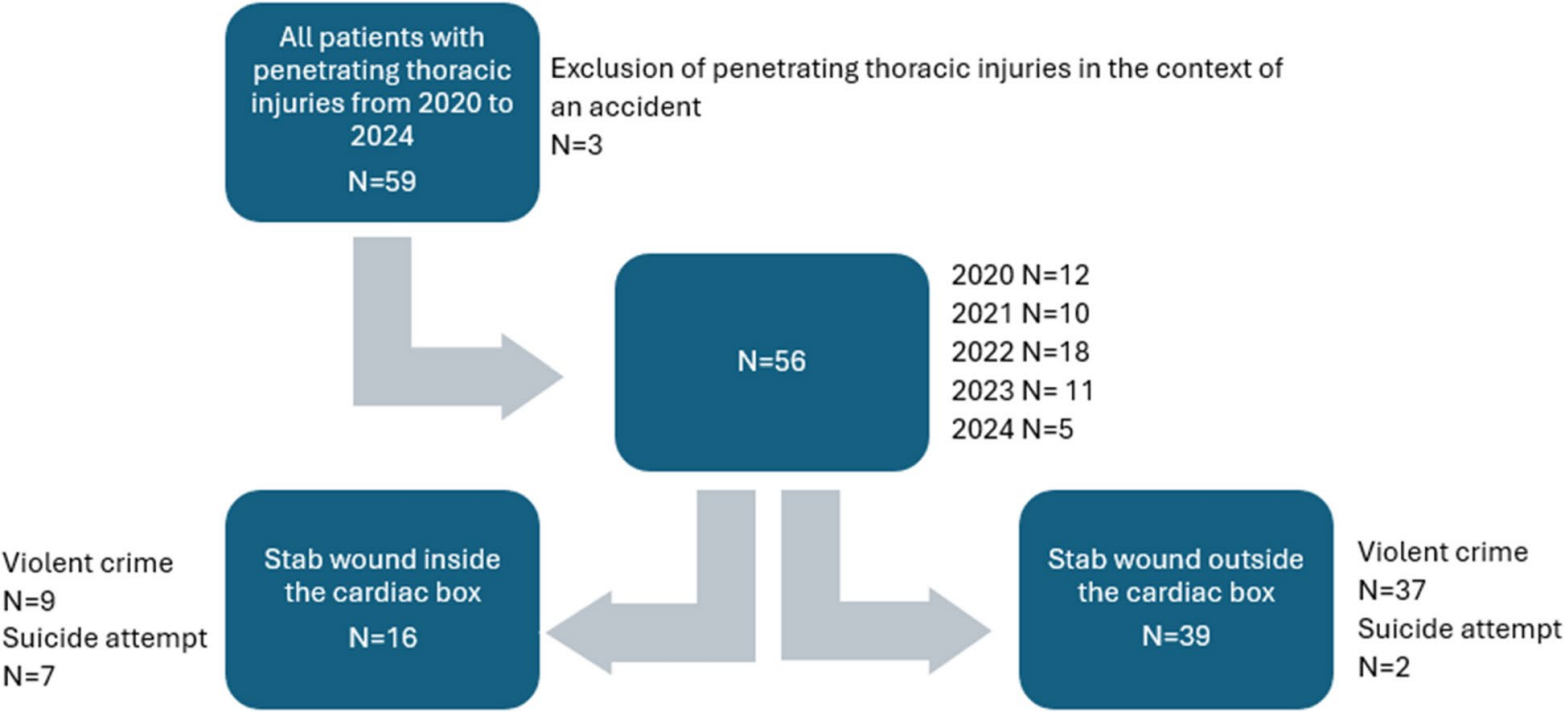
Study cohort. As one patient died before surgery or CT scan classification to one of the groups was not possible

Between January 2020 and June 2024, a total of 56 patients with TSI resulting from assault or self-harm were included in the analysis. Three additional patients with penetrating thoracic trauma caused by occupational or traffic accidents were excluded, as their injuries were predominantly blunt in nature (Fig. 2).

For temporal distribution of patient admissions to the emergency department see Fig. S1 in the Supplementary Section.

Certain statistically significant differences lack clinical relevance and likely reflect random variation due to the limited sample size.

### Weapons characteristics

In all but one case, a knife was the weapon used; the exception involved an assault with a broken glass bottle. The documented blade length ranged from 5 to 30 cm, with information only available for nine patients (16%).

### Demographics

Patient characteristics are summarised in Table 1. All 56 patients were male, with a median age of 28.5 years (interquartile range [IQR] 23.25–36). Over half were aged between 17 and 29 years (n = 29, 51.8%), followed by those aged 30–39 years (n = 14, 25%) and 40–59 years (n = 9, 16%). All patients aged 69 years or older sustained thoracic stab wounds in the context of self-harm (n = 4, 7.2%).

**Table 1:** Patients characteristics

	**Total (n = 56)**	**Inside the cardiac box (n = 16)**	**Outside the cardiac box (n = 39)**	***p*-values**
Male sex (%)	56 (100)	16 (100)	39 (100)	
Age, years (IQR)	28.5 (23.25, 36)	33.5 (23.25, 68.25)	28 (23, 34)	0.061
Attempted suicide (%)	9 (16.1)	**7 (43.8)**	**2 (5.1)**	**0.010**
Violent crime (%)	47 (83.9)	**9 (56.3)**	**37 (94.9)**	0.023
ISS, (IQR)	6 (2, 10)	**9.5 (4, 16.8)**	**3 (1, 9.5)**	0.045
Wound debridement under general anesthesia (%)	40 (71.4)	10 (62.5)	30 (76.9)	0.813
Wound debridement under local anesthesia (%)	5 (8.9)	0 (0)	4 (10,3)	0.646
VATS (%)	8 (14,3)	4 (25)	4 (10,3)	0.241
Thoractomy (%)	2 (3.6)	1 (6.3)	1 (2.6)	0.527
Wedge-resection (%)	3 (5.4)	0 (0)	3 (7.7)	0.083
Operating time, minutes (IQR)	32 (19.25, 58.25)	45 (28, 91)	29 (14, 52)	0.081
Inpatient treatment (%)	52 (92.9)	16 (100)	36 (92.3)	0.900
Outpatient treatment (%)	4 (7.1)	0	3 (7.7)	0.900
Length of hospital stay, days (IQR)	3 (1.5)	4 (1.3, 8)	2 (1.8, 5)	0.168
Postoperative mortality rate (%)	2 (3.6)	**2 (12.5)**	**0 (0)**	0.031

All continuous data are represented as median and interquartile range (IQR) as not stated otherwise. A *p*-value < 0.05 was considered statistically significant. Statistically significant values are highlighted in bold

*ISS* Injury Severity Score

The median age of patients who attempted suicide was significantly higher than that of patients injured through assault (suicide: n = 9, median 53 years [IQR 42–79]; assault: n = 47, median 26 years [IQR 22–33]; p < 0.001).

### Injury localisation and weapon characteristics

Patients were categorised according to the anatomical site of the injury, following the conceptual framework of the cardiac box. Sixteen patients (29%) sustained injuries within the cardiac box (CB^2^ group), whereas 39 (70%) had injuries located outside this area (NCB^3^ group). One patient could not be classified due to missing documentation or imaging.

Injury distribution showed a marked predominance of left-sided wounds (60/77, 78%) compared to right-sided wounds (17/77, 22%). Posterior injuries—defined as those located between the posterior axillary lines—were observed in 29 cases (39%). Sixteen patients (29%) sustained multiple stab wounds, while 40 (71%) had a single thoracic injury (CB: n = 9, 56.3%; NCB: n = 7, 17.9%; *p* = 0.02).

In 51.8% of cases (n = 29), the injury was confined to the thoracic wall without evidence of extension beyond the thorax (CB: n = 8, 50%; NCB: n = 21, 53.8%; *p* = 0.275).

### Resuscitation room management

Overall, 53 patients (94.6%) required management in the resuscitation room (ER^4^). All patients in the CB group were directly admitted to the ER, compared with 94.9% (37/39) in the NCB group. The proportion of ER visits attributable to thoracic stab wounds ranged from 1.4% to 2.4% per year (Fig. S2, Supplementary Section).

According to the Manchester Triage System, 14 patients (87.5%) in the CB group and 32 patients (82.1%) in the NCB group were classified as category “red” (acute life threat). Initial vital parameters and laboratory values are shown in Table 2.

**Table 2:** In-hospital management including clinical findings and laboratory parameters of the initial assessment in the emergency department

	Total (n = 56)	Inside the cardiac box (n = 16)	Outside the cardiac box (n = 39)	*p*-values
Serum lactate, mmol/l (IQR)	2.5 (1.8, 4)	2,7 (2, 3.3)	2,4 (1.4, 4)	0.703
Base excess (IQR)	0.4 (−2, 1.8)	1.1 (−1.9, 2)	−0.1 (−2.1, 1.6)	0.637
Hemoglobin,g/dl, (IQR)	13.8 (12.6, 15.3)	13.5 (12.6, 15.1)	13.8 (12.4, 15.5)	0.999
SpO2,% (IQR)	99 (97, 100)	**97.5 (94.8, 99.3)**	**99 (98, 100)**	0.032
Oxygen demand, %, MV (SD)	29,7 (17.9)	31.8 (17.3)	28.9 (18.3)	0.713
Heart rate, bpm, (IQR)	92,9 (80.8, 105)	92 (75, 102)	93 (82, 107)	0.732
MAP, mmHg (IQR)	96.5 (87.5, 105.3)	93 (81, 110)	97(88, 105)	0.925
GCS, MV (SD)	14 (2,8)	12.9 (4.2)	14.5 (2)	0.351
Vasopressor therapy ER (%)	4 (7,1)	2 (12,5)	2 (5.1)	0.871
Fluid management ER, l, MV (SD)	0.6 (0,7)	0.4 (0, 4)	0.7 (0.7)	0.194
Blood transfusion ER (%)	4 (7.1)	3 (18.8)	1 (2.6)	0.220
Chest tube ER (%)	13 (23.2)	5 (31.3)	8 (20.5)	0.365
Intrahospital transfer time to target station, minutes, (IQR)	51 (38.5, 104.5)	**38 (28, 43)**	**67 (46.5, 130.5)**	< 0.001
Time until surgery, minutes, (IQR)	174 (90, 281)	118 (81, 295)	176.5 (96.8, 280)	0.976

All continuous data are represented as median and interquartile range (IQR) as not stated otherwise. A *p*-value < 0.05 was considered statistically significant

*bpm* beat per minute, *MAP* Mean arterial pressure, *GCS* Glasgow Coma Scale, *ER* Emergency room

**Fig. 3:**
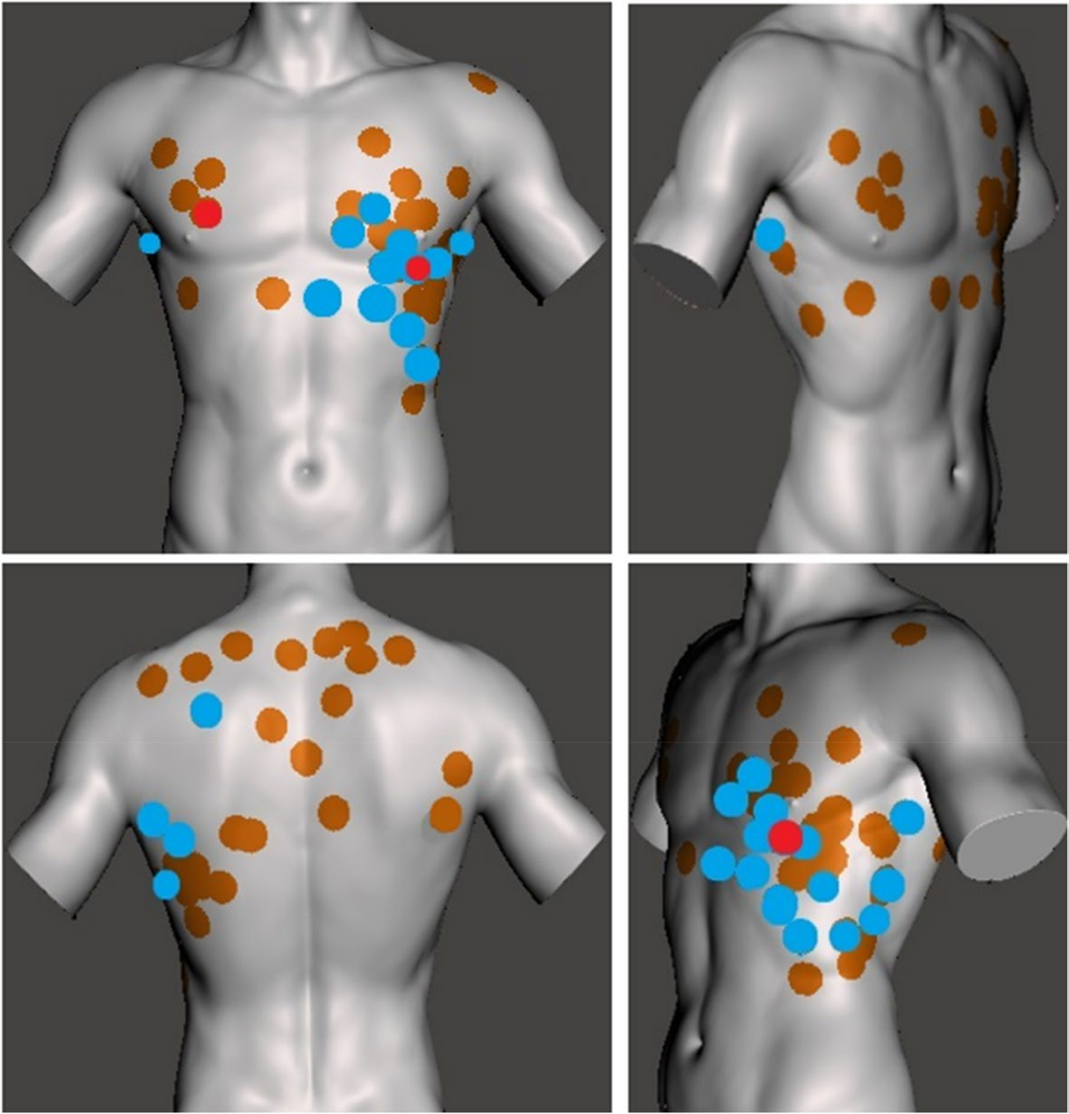
Localisation of the thoracic stab wounds of the study populationblue – patients with thoracic surgery, red – patients with cardiac and/or great vessel injuries. As one patient died before surgery or CT scan, classification to one of the groups (CB or NCB) was not possible

The median Injury Severity Score (ISS) for the cohort was 6 (IQR 2–10), with higher values observed in the CB group compared with the NCB group (CB: median 9.5 [IQR 4–16.8]; NCB: median 3 [IQR 1–9.5]; p = 0.045). Peak median ISS^5^ values were recorded in 2021 and 2024 (ISS 9 for both years). Minor trauma (ISS 1–8) occurred in 28 patients (50%), moderate trauma (ISS 9–15) in 17 patients (30%), and severe trauma (ISS ≥ 16) in 8 patients (14%) (CB: n = 4, 25%; NCB: n = 4, 10%) (Fig. 3).

Two patients in the CB group sustained cardiac and/or great vessel injuries. The sensitivity and specificity of the cardiac box for detecting these injuries were 100% and 73.6%, respectively, with a positive predictive value of 12.5% and a negative predictive value of 100%.

Prehospital intubation was required in six patients (10.7%) (CB: n = 2, 12.5%; NCB: n = 4, 10.3%; p = 0.654). One additional CB patient was intubated upon arrival in the emergency department.

In the prehospital setting, two patients (5.1%) in the NCB group received chest tube placement, whereas none in the CB group did. In the emergency department, chest tubes were placed in 13 patients (23.2%) (CB: n = 5, 31.3%; NCB: n = 8, 20.5%; p = 0.365). An additional 10 patients (17.9%) underwent chest tube insertion intra-operatively (CB: n = 6, 37.5%; NCB: n = 4, 10.3%; p = 0.056).

The mean substitution volume of isotonic crystalloids was slightly lower in the CB group compared with the NCB group (mean ± SD: 0.4 ± 0.4 L vs 0.7 ± 0.7 L; p = 0.194). Vasopressor administration was required in two patients in each group (CB: 12.5%; NCB: 5.1%). The median number of erythrocyte concentrates crossmatched was three (IQR 0–4) in the CB group and two (IQR 0–4) in the NCB group. Transfusion was performed in three CB cases (18.8%) and one NCB case (2.6%) (p = 0.220). Tranexamic acid was administered to five patients (8.9%) (CB: n = 2, 12.5%; NCB: n = 3, 7.7%; p = 0.855). No patient received fresh frozen plasma or prothrombin complex concentrate.

A whole-body CT (WBCT) scan was performed in 18 patients (32.1%) (CB: n = 5, 31.3%; NCB: n = 13, 33.3%; p = 0.884). A trauma-specific CT of the thorax, with or without abdominal imaging, was performed in 33 patients (58.9%) (CB: n = 9, 56.3%; NCB: n = 24, 61.5%; p = 0.948). Two NCB patients (5.1%) received only a chest X-ray.

The median interval between ER arrival and interhospital transfer to the target department (operating theatre, intensive care unit, or general ward) was significantly shorter in the CB group (median 38 min [IQR 38.5–104.5]) compared with the NCB group (median 67 min [IQR 46.5–130.5]; p < 0.001). However, the interval between arrival and commencement of surgery did not differ significantly (CB: 118 min [IQR 81–295]; NCB: 176.5 min [IQR 96.8–280]; p = 0.976).

### Operative management

The majority of patients underwent wound debridement with or without chest tube placement under general anaesthesia (n = 40, 71%). A smaller subset (n = 5, 9%) received wound debridement and closure under local anaesthesia. Consequently, in 80% of cases, no thoracic exploration was required.

Video-assisted thoracoscopic surgery (VATS) was performed in eight patients (14.3%) (CB: n = 4, 25%; NCB: n = 4, 10.3%; p = 0.241). In all cases, the indication for VATS^6^ was the presence of haemothorax and radiological evidence of active bleeding on CT imaging in five patients. Four patients exhibited lung parenchymal injury as the cause for the haemothorax and a wedge resection was performed in three patients (3.6%), all of whom belonged to the NCB group. In one patient the parenchymal injury was detected centrally in the lingula segment of the left upper lobe, bleeding stopped with compression and no resection was performed. In this patient an additional intercostal artery bleeding was stopped using bipolar diathermy. In three of the four patients with parenchymal injury the laceration of the lung was detected on CT imaging preoperatively. Three patients had bleeding caused by a diaphragmatic perforation which was addressed by absorbable, interrupted sutures in each case. All patients who underwent minimally invasive procedures were haemodynamically stable, defined as a systolic blood pressure > 90 mmHg and heart rate < 120 bpm. There was no intra- or postoperative mortality. The mean length of hospital stay in patients undergoing VATS was 4 days.

Two patients (3.6%) required open surgical intervention. In one NCB case, a posterolateral mini-thoracotomy was performed following the diagnosis of haemopneumothorax with intercostal artery rupture. Another patient in the CB group underwent bilateral transverse thoracosternotomy (clamshell thoracotomy) in the trauma bay under continuous cardiopulmonary resuscitation (CPR). A transmural defect of both the anterior and posterior walls of the left ventricle was repaired using single mattress sutures and a Foley catheter placed into the defect. Despite prolonged resuscitative efforts, the patient did not achieve return of spontaneous circulation and died intraoperatively. A second patient in the CB group died in the emergency department during CPR before surgical intervention could be initiated.

The distribution of thoracic injuries is presented in Table 3. The median operative duration was longer in the CB group (45 min, IQR 28–91) compared with the NCB group (29 min, IQR 14–52), although this difference did not reach statistical significance (p = 0.081). During surgery, two patients in each group required red blood cell (RBC) transfusion (CB: 12.5%; NCB: 5.1%; p = 0.569).

**Table 3:** Range of the exclusively thoracic injuries depending on the localisation of the stab wound

	Total (n = 56)	Inside the cardiac box (n = 16)	Outside the cardiac box (n = 39)	*p*-values
Hematothorax (%)	5 (7.9)	2 (12.5)	3 (7.7)	0.622
Hematopneumothorax (%)	9 (14.3)	3 (18.8)	6 (15.4)	0.71
Pneumothorax (%)	5 (7.9)	3(18.8)	2 (5.1)	0.141
Tension pneumothorax (%)	1 (1.6)	0 (0)	1 (2.6)	1
Pneumomediastinum (%)	2 (3.2)	1 (6.3)	1 (2.6)	0.501
Hemopneumoperikardium (%)	1 (1.6)	1 (6.3)	0 (0)	0.291
Lung parenchyma injury (%)	6 (9.5)	2 (12.5)	4 (10.3)	1
Diaphragm perforation (%)	6 (9.5)	4 (25)	2 (5.1)	0.053
Ventricle perforation (%)	1 (1.6)	1 (6.3)	0 (0)	0.291
Intercostal artery rupture(%)	3 (4.8)	2 (12.5)	1 (2.6)	0.199
Rib fracture (%)	2 (3.2)	0 (0)	2 (5.1)	1
Singular extrapleural injury (subcutaneous and/or muscular injury) (%)	21 (33.3)	3 (18.8)	18 (46.2)	0.073

A *p*-value < 0.05 was considered statistically significant

### Postoperative care and outcome

Most patients required hospital admission (n = 52, 92.9%), while four patients were treated as outpatients—all of whom belonged to the NCB group. The median length of hospital stay was slightly longer in the CB group (median 4 days [IQR 1.8–8]) compared with the NCB group (median 2 days [IQR 1.8–5]; p = 0.168).

Major postoperative complications (≥ Grade II, Clavien–Dindo classification) were significantly more frequent in the CB group (50.1%) compared with the NCB group (25.7%; p = 0.018). The overall mortality rate was 3.6% (n = 2), with both fatalities occurring in the CB group.

## Discussion

The cardiac box concept was first described in 1967 by Sauer and Murdock at the Mobile General Hospital in Alabama, USA, as a means to identify an “effective and expeditious manner” of managing penetrating injuries to the heart and great vessels [7]. Their definition encompassed a “danger zone” bordered cranially by the suprasternal notch, laterally by the midlines of the left and right clavicles, and caudally by the epigastrium and sixth intercostal spaces. Subsequent studies expanded these boundaries, occasionally extending them to the posterior thoracic wall, leading to variations in its application across international studies [13–16]. Cardiac injuries have been reported in 60–84% of cases when the wound is located within the cardiac box [13, 14].

However, the validity of this anatomical concept has long been debated. Siemens et al. were among the first to demonstrate that conservative management could be appropriate in selected cases [11]. Demetriades et al. subsequently argued that “the presence of a wound over the precordium is not in itself an absolute indication for surgery” [17]. Degiannis et al. found that extra-precordial stab wounds were associated with higher mortality than precordial ones (25% vs 4%), likely due to diagnostic delays [18]. Jhunjhunwala et al., in a study of gunshot wounds, observed that left-sided thoracic injuries were most frequently associated with cardiac involvement, suggesting that the cardiac box is inadequate as a sole predictor of such injuries [19]. Kim et al. similarly reported a higher incidence of cardiac injury and thoracotomy in patients with wounds within the cardiac box but no significant difference in mortality [10]. In our series, cardiac and/or great vessel injuries occurred exclusively in patients with wounds inside the cardiac box. Both patients died resulting in a mortality rate of 12.5% in this subgroup. Although this supports a potential association, the predictive value of the cardiac box was low (positive predictive value 12.5%; negative predictive value 100%). Furthermore, the distribution of intrathoracic injury patterns was similar between groups, with lung parenchymal resection required only among NCB patients. While emergency thoracotomy rates did not differ by injury location, intrathoracic exploration was more frequently undertaken in the CB group. In contrast to gunshot wounds, the majority of stab wounds that cause cardiac injuries (80%) are in the precordial area [20, 21]. Kleber et al. also demonstrated that 60% of patients with penetrating chest injuries showed to have an isolated cardiac injury or a combination with thoracic vascular injury in his assessment on prehospital and preventable trauma-related deaths in Berlin in 2010 [22]. Yet, in our cohort the positive predictive value of cardiac box injury was modest (12.5%) and the majority of patients with stab wounds inside the cardiac box had no cardiac or great vessel injuries. Of note, no patient with TSI outside the cardiac box suffered from a cardiac injury resulting in a high negative predictive value for cardiac box injuries. Moreover, the distribution of intrathoracic injury patterns were similar in both CB and NCB groups although lung parenchymal injury with the need for resection was only performed in the NCB group. Whilst the absolute level of emergency thoracotomy did not differ regarding injury localization, a greater proportion of patients underwent surgery for intrathoracic exploration when the injury was located inside the cardiac box.

Seamon et al. reported that survival after emergency department thoracotomy for penetrating thoracic trauma depended not solely on anatomical injury site, but on the mechanism of injury, the presence of vital signs upon admission, and the number of cardiovascular structures affected [23]. Their analysis showed higher survival for stab wounds (24.2%) than gunshot wounds (4.8%), and for single compared with multiple cardiac or great vessel injuries. Given that stab wounds typically involve low-energy transfer, the trajectory and blade length are crucial determinants of injury severity. Violent assault was the predominant mechanism of injury in our cohort. However, the study’s design does not permit direct comparison with police-recorded statistics, as our focus was restricted to hospital-based data. Notably, all patients who sustained self-inflicted stab injuries were aged over 69 years, consistent with national data showing an age-dependent rise in suicide incidence [24]. In our series, 78% of suicide attempts involved injuries within the cardiac box, highlighting a high potential for cardiac or major vascular involvement. Furthermore, the cardiac box group includes a markedly higher proportion of self-inflicted stab wounds (43.8%) compared with the non-cardiac box group (5.1%), while violent assaults predominate in the latter (94.9%). Comparable findings have been reported in an Australian analysis of self-inflicted stab wounds, in which 73% of single-stab suicides involved the chest, predominantly on the left side, reflecting a particularly mechanism for cardiac injuries and/or the great vessels [25]. Moreover, a Swedish forensic study of the manner of death in single stab injuries to the trunk showed a predomination for suicidal stabs for the anterior and left thoracic wall whereas stabs in homicides are usually more lateral [26].

Our findings also suggest heightened clinical awareness among attending physicians regarding potentially life-threatening injuries associated with the cardiac box. This may explain the shorter transfer times to the operating theatre or target department in the CB group, as well as the higher frequency of “red” triage classifications. However, this raises the question of whether the cardiac box’s role as a prehospital and early in-hospital indicator of critical injury risk may be overestimated. It may therefore be argued that, in the prehospital phase, injuries within the cardiac box should prompt rapid transfer to a cardiothoracic surgical centre. However, our data suggest that such consultation is seldom required, as most patients do not sustain cardiac or great vessel injury. Stranch et al. similarly demonstrated that acute care surgeons could manage penetrating thoracic trauma—including cardiac box injuries—safely and effectively, without the need for cardiothoracic consultation or cardiopulmonary bypass [27]. A plausible explanation is the prehospital mortality of severe cardiac injuries, as only 6–25% of patients with such injuries reach hospital alive [28–30]. Among those who do, 75–80% are haemodynamically stable on arrival [1], consistent with our cohort, in which only four patients required vasopressors or transfusion support.

Prehospital chest drain insertion was performed in only 3.6% of patients in our study, whereas an additional 23.3% required drainage upon arrival in the emergency department. Bieler et al. reported a similar rate (6%) in the TraumaRegister DGU® cohort (2009–2018) [1], with approximately 40% of prehospital chest drains later deemed inadequate. The impact on mortality and the rationale behind this relatively low proportion of prehospital chest drains remain to be elucidated. Ondruschka et al. documented that in 30% of trauma deaths with prehospital resuscitation (tCPR), pleural decompression was either incorrectly performed or omitted altogether [31]. An online survey of German prehospital emergency physicians revealed that only one-third performed chest drains regularly, and one-quarter felt insecure in the procedure, whereas clinical experience and simulation training improved confidence [32]. The S3 Guideline on Polytrauma and Severe Injury Management recommends immediate decompression—via needle or mini-thoracotomy, with or without chest drain placement—in suspected tension pneumothorax [33]. For spontaneously breathing patients, a pneumothorax can be treated conservatively with close clinical observation. The low frequency of prehospital decompression observed in our cohort underscores the need for enhanced procedural training, including simulation-based education and clear decision-making algorithms, as failure to decompress a tension pneumothorax remains one of the leading preventable causes of death in traumatic cardiac arrest [34, 35]. Our findings therefore suggest relevant gaps in guideline adherence, which may stem from limited procedural exposure, insufficient training opportunities, or uncertainties in prehospital decision-making.

In 9% of cases, an intra-abdominal injury accompanied an isolated thoracic stab wound (CB: n = 2; NCB: n = 3), highlighting the importance of considering blade length and trajectory, particularly in the thoracoabdominal transition zone. Approximately one-quarter of thoracic stab wounds have been reported to involve the abdomen, and conversely, up to 60% of penetrating abdominal injuries affect the thorax [1]. The high incidence (39%) of posterior thoracic wounds in our cohort underscores the importance of performing a complete log-roll manoeuvre during trauma room assessment. Notably, documentation of blade length in both prehospital and in-hospital records was frequently missing, likely reflecting the chaotic nature of the incident scene, absence of witnesses, or language barriers. Improved documentation could enhance injury assessment and medico-legal accuracy.

Once in-hospital management begins, diagnostic imaging—particularly extended focused assessment with sonography for trauma (eFAST)—assumes a pivotal role. eFAST demonstrates high diagnostic accuracy, with reported sensitivity and specificity of 79% and 93%, respectively, for detecting occult cardiac injuries in stable patients [36, 37]. Several authors have suggested that eFAST may provide superior decision-making support compared with anatomical localisation alone [10, 38, 39].

However, these findings reflect a single-center experience of a Level 1 Trauma Center in Germany and given the small sample size generalisation should be made with caution. Likewise, in other European Countries the incidence of TSI is similar low but in the context of increasing knife related injuries all over Europe this topic remains highly relevant for emergency medicine [40–42].

The principal limitations of our study include the small sample size and retrospective and exploratory single-centre design, which restrict generalisability. Considering this and multiple subgroup analyses, our findings should be interpreted as exploratory, and the potential risk of type I error cannot be excluded. Our analysis cannot account for prehospital mortality, and while efforts were made to obtain police data, additional information was unavailable. The rarity of penetrating thoracic trauma in Germany necessarily limits cohort size and results in a primarily descriptive analysis but may reflect practice in many European centres.

## Conclusion

In conclusion, this single-centre analysis of penetrating thoracic injuries over nearly four years demonstrates a consistently low incidence, predominantly related to violent assault or self-harm. A minimally invasive, parenchyma-sparing surgical approach appears feasible in haemodynamically stable patients requiring exploration, whereas open procedures remain indicated for unstable cases requiring emergency thoracotomy. The paucity of prehospital documentation regarding blade length represents a notable limitation in injury assessment. Furthermore, the low rate of prehospital chest drain placement warrants further investigation into training practices and clinical decision-making. Finally, our findings suggest that the cardiac box concept, while intuitively useful, may be overly simplistic and insufficient as a predictor of cardiac or great vessel injury. Injuries outside this anatomical zone may still be life-threatening and should not be underestimated during clinical assessment.

## Supplementary Information

Supplementary Material 1.

## Data Availability

The datasets used and/or analysed during the current study are available from the corresponding author on reasonable request.

## Footnotes

1 Thoracic stab injury/injuries.

2 CB – Cardiac Box group.

3 NCB – Non Cardiac Box group.

4 ER – Emergency room.

5 ISS – Injury Severity Score.

6 VATS – Video-assisted Thoracic Surgery.
