# Supplementary material for: Penetrating thoracic stab wounds and the cardiac box: a single-center experience of in-hospital treatment and outcome in Germany
Source: Scand J Trauma Resusc Emerg Med. 2026 Jan 17;34:18. doi: 10.1186/s13049-026-01555-y (PMC12849326; doi:10.1186/s13049-026-01555-y)
Supplement: Supplementary file 1 — Supplementary Material 1. [file 13049_2026_1555_MOESM1_ESM.docx]

***Supplementary Section***

**Figure S1** Distribution of the arriving time at the emergency department.

**Figure S2** a) Annual proportion of patients with TSI in the emergency room (ER) among all patients treated in the ER b) Annual proportion of patients with TSI among all patients treated in the emergency department from 2020 until 06/2024

a)

b)
